
JOURNAL INFORMATION
==============================
NLM Title Abbreviation: ScientificWorldJournal
Journal ID: ScientificWorldJournal
NLM Journal ID: 101131163
Journal ID: TSWJ

The Scientific World Journal

ISSN: 2356-6140
EISSN: 1537-744X
Publisher: Wiley

ARTICLE INFORMATION
==============================
PMCID: PMC6084685
PMID: 30147527
DOI: 10.1100/tsw.2001.228
Article ID: 543808
Article version: 1
Subjects: Short Report

Purine Inhibitors of Cyclin-Dependent Kinase and Their Apoptosis and Cytotoxicity Mechanisms

Strnad M. 1 *
Havlíček L. 1
Kryštof P. V. 1
Lenobel R. 1
Hanuš J. 1
Siglerová V. 1
Hajdúch L. M. 2
1 Laboratory of Growth Regulators, Institute of Experimental Botany ASCR & Palacký University, Šlechtitelů 11, 783 71 Olomouc, Czech Republic
2 Laboratory of Experimental Medicine, Palacký University, Hněvotínská 3, 775 15 Olomouc, Czech Republic
*M. Strnad: strnad@risc.upol.cz
Publication date: 2001
Electronic publication date: 2001 Apr 4
Volume: 1
First page: 58
Last page: 58
Copyright: Copyright © 2001 M. Strnad et al.
Copyright year: 2001
License: This is an open access article distributed under the Creative Commons Attribution License, which permits unrestricted use, distribution, and reproduction in any medium, provided the original work is properly cited.
License URL: https://creativecommons.org/licenses/by/3.0/

Funding: Ministry of Education, Youth and Sports MSM 153100008

==============================
Miami *Nature Biotechnology* Short Reports
*TheScientificWorld* (2001) 1 (S3), 125SR
ISSN 1532-2246; DOI 10.1100/tsw.2001.228

### PURINE INHIBITORS OF CYCLIN-DEPENDENT KINASE AND THEIR APOPTOSIS AND CYTOTOXICITY MECHANISMS

M. Strnad[1*], L. Havlíček[1], P.V. Kryštof[1], R. Lenobel[1], J. Hanuš[1], V. Siglerová[1], L.M. Hajdúch[2]

[1]Laboratory of Growth Regulators, Institute of Experimental Botany ASCR & Palacký University, Šlechtitelů 11, 783 71 Olomouc; [2]Laboratory of Experimental Medicine, Palacký University, Hněvotínská 3, 775 15 Olomouc, Czech Republic
* strnad@risc.upol.cz

**INTRODUCTION.** The importance of cell cycle regulatory proteins, their direct interaction with oncogenes and the tumour suppressor pRb and their frequent deregulation in human tumours has encouraged an active search for low-molecular weight regulators of these proteins. Among them the chemical inhibitors of cyclin-dependent kinases were the first discovered. These inhibitors are anti-mitotic and display very interesting anti-tumour activities.

**METHODS.** Chemical synthesis was based on alkylation of 2,6-dichloropurine. Newly developed compounds were then tested in different CDK assays (1). Antiproliferative, cytotoxic and pro-differentiating capacity of olomoucine and its structural derivatives on the range of human and animal cell lines. We have been using the following cell lines: *HELA* (human cervical carcinoma), *MCF7* (human breast adenocarcinoma), *NIH 3T3* (mouse fibroblasts), *HOS* (human osteogenic sarcoma), *HL 60* (human promyelocytic leukemia), *G 361* (human malignant melanoma), *K562* (human erythroleukaemia), *CEM* (human lymphoblastoid leukaemia). Tested drugs were added to the cell cultures in six different concentrations and kept at 37°C and 5% $CO_2$ for three days. After that, viability of cells in cultures was examined by calcein AM assay. The concentration killing 50% of tumour cells will be calculated.

**RESULTS.** Our research focused on the primary mechanism of action of plant hormones cytokinins ($N^6$-substituted adenine derivatives) in cell division cycle has showed that natural plant cytokinins are rather non-specific inhibitors of various protein kinases (2). Surprisingly, among aromatic cytokinin derivatives, we have discovered a compound, 2-(2-hydroxyethylamino)-6-benzylamino-9-methylpurine, named "olomoucine" (OC, Fig. 1), which specifically inhibits some cyclin-dependent kinases (CDKs) at micromolar concentration (2). One of the inhibited kinases, the $p34^{cdc2}$/cyclin B kinase, assumed to be a key mitotic factor, which is highly conserved and strongly implicated in cell cycle transitions in all eukaryotic cells (3). The total lack of the inhibitory effect of olomoucine on major kinases, such as cAMP- and cGMP-dependent kinases, protein kinase C, and others, suggests that OC might be a useful tool for cell cycle regulation studies. The design and inhibitory activity of OC was further improved by modifications at positions 2, 6,and 9, i.e., the positions that control binding to CDK1. This has recently led to discovery of novel specific CDK inhibitor named roscovitine, purvalanol A and B, etc. (1,4; Fig. 1), which displays an enhanced inhibitory activity toward CDK1, a higher selectivity toward some CDKs, an increased antimitotic activity at the G1/S and G2/M points of the cell cycle, and stronger and more selective antitumor effects. Finally, we have tested different CDK inhibitors on several tumour cell lines (CEM, HL 60, etc.). The average concentration that causes 50% growth inhibition is 2-100 μM.

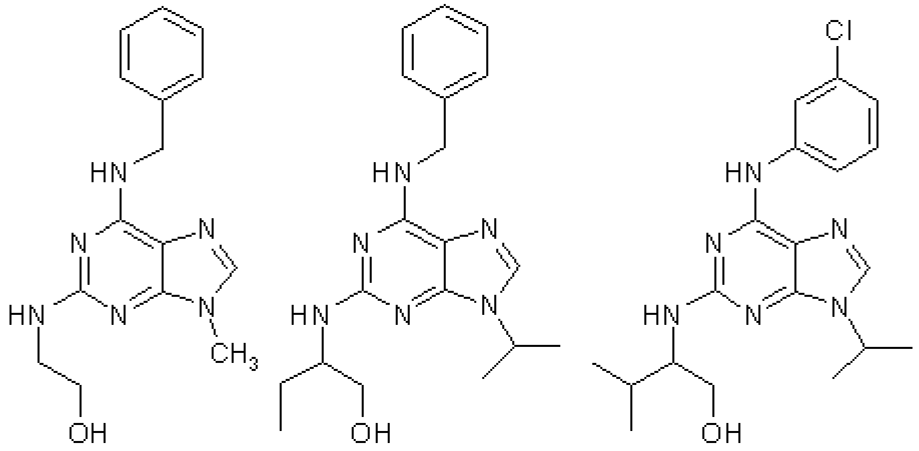

**Fig. 1.** Chemical structures of olomoucine, roscovitine and purvalanol A (from left to right).

**DISCUSSION.** Being stimulated by discovery of olomoucine many other groups have started to synthesise purine-derived CDK inhibitors (5,6). There are also other types of CDK inhibitors structurally unrelated with the trisubstituted purines.

**ACKNOWLEDGEMENT.** This work is supported Czech Ministry of Education via grant MSM 153100008.

**REFERENCES.**

1. Havlíček, L., Hanuš, J., Veselý, J., Leclerc, S., Meijer, L., Shaw, G., and Strnad, M. (1997) J. Med. Chem. 40, 408-412
2. Veselý, J., Havlíček, L., Strnad, M., Blow, J.J., Donella-Deana, A., Pinna, L., Letham, D.S., Kato, J., Detivaud, L., Leclerc, S., and Meijer, L. (1994) Eur. J. Biochem. 224, 771-786
3. Harper, J.W. and Elledge, S.J. (1996) Curr. Opin. Genet. Dev. 6, 56-64
4. De Azevedo, W.F., Leclerc, S., Meijer, L., Havlíček, L., Strnad, M., and Kim, S.H. (1997) Eur. J. Biochem. 243, 518-526
5. Gray, N., Détivaud, L., Doering, C., and Meijer, L. (1999) Curr. Med. Chem. 6, 859-875
6. Silecki, T.M., Boylan, J.F., Benfield, P.A., and Trainor, G.T. (2000) J. Med. Chem. 43, 1-18
